# Triglyceride Level Is an Independent Risk Factor in First-Attacked Neuromyelitis Optica Spectrum Disorders Patients

**DOI:** 10.3389/fneur.2019.01230

**Published:** 2019-11-21

**Authors:** Kaimin Wu, LuLu Wen, Ranran Duan, Yanfei Li, Yaobing Yao, Lijun Jing, Yanjie Jia, Junfang Teng, Qianyi He

**Affiliations:** Department of Neurology, The First Affiliated Hospital of Zhengzhou University, Zhengzhou, China

**Keywords:** neuromyelitis optica spectrum disorders, the first-attacked patients, triglyceride level, outcomes, relapse

## Abstract

**Objective:** To investigate prospective associations between triglyceride (TG) level and prognosis of first-attacked patients with neuromyelitis optica spectrum disorders (NMOSD).

**Methods:** This retrospective study included 196 patients newly diagnosed with NMOSD from June 2014 to December 2018. Data of clinical parameters, including age of onset, sex, BMI, blood lipid levels, anti-aquaporin-4 status, serum glucose level, therapy regimens, comorbidities, initial Expanded Disability Status Scale (EDSS), relapses, and outcomes were collected. We used logistic regression models to examine the associations among relevant clinical factors and outcomes, and statistical analyses were performed using the SPSS 23.0 software.

**Results:** Compared with the high TG group, residual EDSS was relatively lower in the normal TG group (median 1.0 vs. 2.0, *P* = 0.002). In the univariate analysis, TG level was positively correlated with outcomes (OR 1.75, 95% CI 1.18–2.60, *P* = 0.005) and relapses (OR 1.57, 95% CI 1.07–2.31, *P* = 0.02). Our stratified analysis suggested that patients with normal BMI (OR 4.90, 95% CI 2.10–11.44, *P* = 0.001) were closely correlated with poor recovery owing to increased TG level. In the multivariate analysis, a statistically significant association still existed between TG level and outcomes (OR 3.44, 95% CI 1.02–11.64; *P* = 0.040) after adjusting for various variables.

**Conclusions:** In first-attacked NMOSD patients, TG level was positively associated with poor recovery. Early monitoring and treatment of elevated TG level in NMOSD patients are important.

## Introduction

Neuromyelitis optica spectrum disorders (NMOSD) is a type of autoimmune demyelinating disease that results in inflammatory lesions in the optic nerves, spinal cord, and other areas of the central nerve system (CNS) (1). Although NMOSD is a rare disorder, it can cause serious disability partly because of high recurrence risk and progressive disability (2). In the last decade, efforts have been made to understand the potential pathogenic mechanisms and identify modifiable risks factors to explore effective preventative measures to cure this disease. Approximately 80% NMO cases are seropositive for aquaporin-4 IgG (3), and myelin oligodendrocyte glycoprotein IgG is also a highly specific diagnostic marker for NMOSD (4). Besides these autoantibodies, many other factors, such as vitamin D level (5), hormone level (6), gastrointestinal infection (7), pregnancy (8), and diet (9), are also involved in the pathological process of NMOSD.

Dyslipidemia, a common comorbidity of cardiovascular diseases (10), has been proposed to be related to the onset and progression of various autoimmune diseases. The values of total cholesterol (TC), low-density lipoprotein (LDL), and triglyceride (TG) during the flare period of systemic lupus erythematosus were all higher than those in the remission stage (11). A large-scale observational research found that higher TC level was linked to worsening disability in those suffering from multiple sclerosis (MS) (12). There were also a positive association between TC levels and disability scores in patients with relapsing-remitting form of MS (13). Another research suggested that high TG level increases the probability of recurrence of MS (14).

There are limited studies on whether lipid-related variables affect disease progression and prognosis in patients with NMOSD. This study aimed to assess the association between lipid profile (especially TG level) and outcomes in patients newly diagnosed with NMOSD.

## Materials and Methods

### Patients

**Figure 1:**
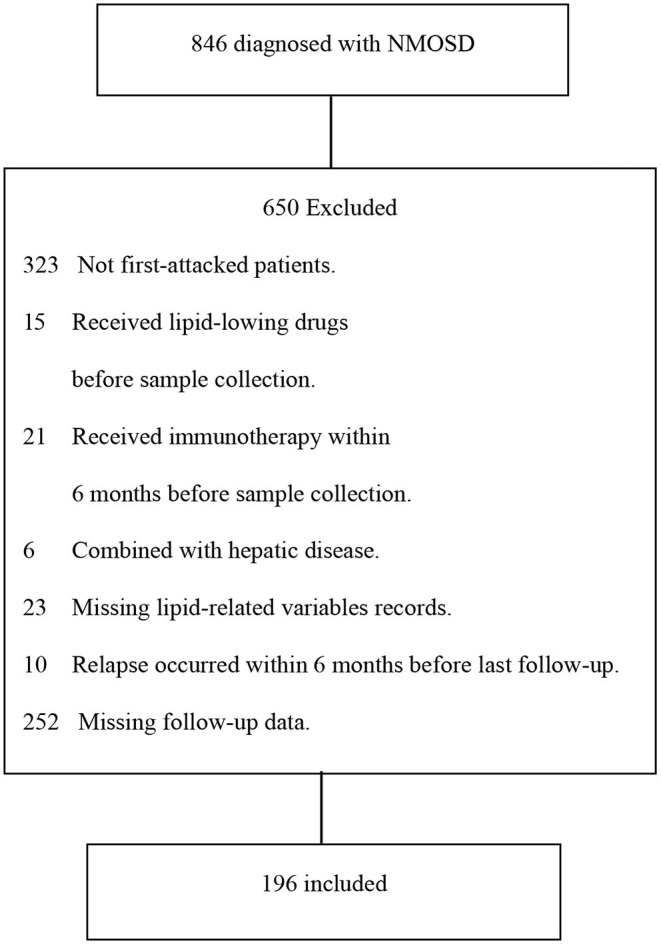
Patients screening process. From June 2014 to December 2018, 846 consecutive cases with NMOSD were admitted to our department. Six hundred and fifty patients did not meet the inclusion criteria, and we finally included 196 patients.

Between June 2014 and December 2018, 846 consecutive patients admitted to the First Affiliated Hospital of Zhengzhou University and newly diagnosed with NMOSD participated in this cohort study. All patients fulfilled the latest diagnostic criteria for NMOSD (1). Inclusion criteria for the NMOSD cohort were as follows: (A) enrolled cases must be confirmed as the first-attacked NMOSD patients by a professional expert of neurology, (B) without hepatic disease, (C) subjects were not prescribed lipid-lowering drugs prior to admission, but those who received statin treatment during the course of this disease were included. (D)patients did not receive immunomodulatory treatment within 6 months prior to admission, and (E) patients who relapsed within 6 months before last follow-up or with incomplete data were excluded. The participants selection process is shown in Figure 1.

### Data Collection

Medical records of all subjects were retrospectively reviewed. Baseline clinical information including age of onset, sex, height, weight, blood lipid levels, anti-aquaporin-4 (AQP4) status, serum glucose level, clinical symptoms, therapy regimens, and comorbidities were collected. Expanded Disability Status Scale (EDSS) at the time of the first attack was carefully evaluated by a professional neurologist and recorded as initial EDSS. Baseline EDSS before the first attack was considered to be normal in all cases.

### Biomarker Measurement

The analysis of blood samples was performed at the Biochemistry Laboratory of the First Affiliated Hospital of Zhengzhou University. When more than one fasting lipid test was performed during hospitalization, data from the sampling closest to admission were considered. Fasting TC, LDL, HDL, TG, and glucose levels were evaluated using enzymatic kits (Sigma-Aldrich). All testing was carried out in accordance with the manufacturer's protocols and the examiners were blinded to diagnoses or clinical symptoms.

### Anti-AQP4 Status

Serum or cerebrospinal fluid samples were used to detect anti-AQP4 status at the Neurology Laboratory of the First Affiliated Hospital of Zhengzhou University, using an assay on live cells transfected with AQP4.

### Outcomes

Clinical outcomes in patients at the last follow-up were recorded as residual EDSS. Relapse events and residual EDSS score in last follow-up were obtained during clinic visits or via telephone interview. To understand the associations between TG level and clinical outcomes, the residual EDSS variable was rendered into categorical variables. The subjects were divided into two groups according to the residual EDSS scores. We defined subjects with residual EDSS scores above 3 (EDSS > 3) as patients with poor recovery, and the rest were defined as patients with good recovery. Relapses were defined as per the 2001 McDonald criteria as the new onset or recurrent neurological symptoms (lasting for at least 24 h) in the absence of fever or infection (15), and the interval time referred to the time to first relapse.

### Statistical Analysis

Data analysis was conducted using the SPSS 23.0 software (International Business Machines Corporation, Chicago, IL, USA). Continuous data were presented as the mean ± SD if the Kolmogorov–Smirnov test showed that they were normally distributed; otherwise, the data were expressed as median (IQR). Comparisons of means between continuous demographic variables were performed by *t*-test or Kruskal–Wallis test. Categorical variables were indicated in terms of frequency (percentage, %) and compared using the chi-square or Fisher's exact test. Univariate logistic regression models were used to identify whether TG level or other covariates had an independent effect on outcomes. The covariates are as follows: age of onset, sex, BMI, glucose level, TC, TG, LDL, HDL, AQP4 status, initial EDSS, autoimmune diseases, hypertension, diabetes, therapeutic regimens (including steroids, immunosuppressive therapy, rehabilitation training, and statin treatment), and relapses. Interaction and stratified analysis were employed to examine the association between TG level and the outcomes according to variables mentioned above and continuous data were grouped into dichotomous variables, including age (≤ 60 and > 60 year), BMI (≤ 25 and > 25 kg/m^2^) (16), TC (≤ 5.0 and > 5.0 mmol/L), HDL (≤ 1.0 and > 1.0 mmol/L), LDL (≤ 3.0 and > 3.0 mmol/L) (17), Glucose (≤ 6.1 and > 6.1 mmol/L) (18), and Initial EDSS (≤ 3 and > 3). For multivariate logistic regression, we adjusted multiple confounders to analyze the stability of the associations between TG level and outcomes. Variables, including TC, LDL, and HDL, were considered in the basic model, because lipoproteins are all involved in lipid metabolism. Previous studies demonstrated that age of onset, sex, BMI, statin treatment, initial EDSS, and relapse were significantly associated with outcomes in NMOSD (19–21); hence, Adjust-I model further adjusted for these factors. We finally examined the associations between TG level and outcomes by furtherly adjusting for glucose levels, treatment methods, and complications in Adjusted II model. The significance level was established at *p* < 0.05.

## Results

### Demographic and Clinical Characteristics

**Figure 2:**
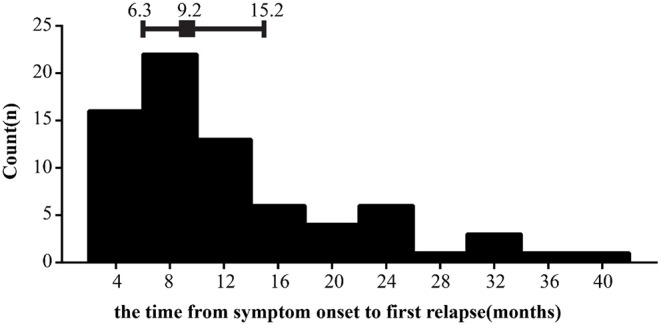
Distributions of the interval time from symptom onset to first relapse. Error bars show median surrounded by 25th and 75th percentiles.

In this cohort study, a total of 196 cases with newly diagnosed NMOSD were enrolled and the detailed screening process is shown in Figure 1. The demographic and clinical characteristics of all patients are presented in Table 1. To better understand the effect of TG level on clinical prognosis, we classified patients into two groups according to serum TG level: normal TG group (TG ≤ 1.7 mmol/L) and high TG group (TG >1.7 mmol/L). Compared with the high TG group, levels of TC were dramatically lower (median 4.3 vs. 5.0, *P* = 0.005), and those of TG also remarkedly decreased (median 1.0 vs. 2.3, *p* < 0.001), while those of HDL significantly increased in the normal TG group (median 1.3 vs. 1.1, *P* = 0.003). Other parameters, including age at onset, ratio of female patients, BMI, LDL and glucose levels remained unchanged. Clinical manifestations of all 196 patients were carefully evaluated at first attack and recorded as initial EDSS, but no significant differences were observed between the two groups. Only half the patients were tested for anti-AQP4 status in cerebrospinal fluid or serum, and the positive results in both groups were more than 60%. There were also no significant differences in the number of patients who suffered from hypertension, diabetes or autoimmune diseases (including systemic lupus erythematosus, sicca syndrome, IgA nephropathy, and thyroiditis). Considering clinical symptoms and financial situations, patients received different treatments, such as corticosteroids, immunosuppressants (azathioprine, rituximab, and mycophenolate mofetil), rehabilitation training, and statin treatment, but no significant differences emerged between the two groups. At the last follow-up, the residual EDSS scores were significantly lower in the normal TG group than in the high TG group (median 1.0 vs. 2.0, *P* = 0.002), and the proportion of patients who gained a good recovery was higher in the normal TG group than in the high TG group (78.2 vs. 50.0%, *P* = 0.006). Approximately 36% of patients relapsed during the follow-up period, and the interval time was left skewed with a median time 9.2 months (Figure 2), but relapses rate and the interval time between two groups during follow-up were not markedly different.

**Table 1:** Characteristics of the patients with first attacked NMOSD according to TG group.

**Clinical characteristics**	**Total patients**	**Normal-TG group**	**High-TG group**	***P*-value**
	**(*n* = 196)**	**(*n* = 156)**	**(*n* = 40)**	
Age of onset, years, mean ± SD	43.4 ± 13.6	43.1 ± 14.8	45.1 ± 13.9	0.43
Sex, female, *n* (%)	124 (63.3)	99 (63.5)	25 (62.5)	0.91
BMI, (Kg/m^2^), median (IQR)	22.9 (20.4–25.4)	22.8 (20.3–25.3)	23.1 (20.6–25.4)	0.86
TC, median (IQR), mmol/L	4.3 (3.7–5.0)	4.3 (3.6–4.9)	5.0 (4.0–5.7)	0.005^*^
TG, median (IQR), mmol/L	1.2 (0.8–1.6)	1.0 (0.8–1.3)	2.3 (1.9–4.1)	<0.001^*^
HDL, median (IQR), mmol/L	1.2 (1.0–1.4)	1.3 (1.0–1.4)	1.1 (0.9–1.2)	0.003^*^
LDL, mean ± SD, mmol/L	2.7 (2.2–3.4)	2.7 ± 0.6	3.0 ± 1.2	0.15
Glucose, median (IQR), mmol/L	4.7 (4.2–5.7)	4.9 (4.2–5.8)	4.4 (4.2–5.8)	0.55
Initial EDSS, median (IQR)	4.0 (3.0–6.0)	4.0 (3.0–6.0)	5.0 (3.8–6.5)	0.12
Anti-AQP4 status, *n* (%)				
Negative	29 (31.2)	22 (29.7)	7 (36.8)	0.53
Positive	64 (68.8)	52 (70.3)	12 (63.2)	0.99
Hypertension, *n* (%)	14 (7.1)	10 (6.4)	4 (10.0)	0.49
Diabetes, *n* (%)	9 (4.6)	7 (4.5)	2 (5.0)	0.69
Autoimmune diseases, n (%)	10 (5.1)	8 (5.1)	2 (5.0)	0.97
Corticosteroid treatment, n (%)	180 (91.8)	143 (91.7)	37 (92.5)	0.86
Immunosuppressant treatment, *n* (%)	56 (28.6)	42 (26.9)	14 (35.0)	0.31
Rehabilitation training, *n* (%)	39 (19.9)	30 (19.2)	9 (22.5)	0.64
Statin treatment, *n* (%)	21 (13.5)	15 (9.9)	6 (15%)	0.48
Residual EDSS, median (IQR)	1.5 (0.5–3.5)	1.0 (0–3.0)	2.0 (1.8–4.0)	0.002^*^
Outcomes, good recovery, *n* (%)	142 (72.4)	122 (78.2)	20 (50.0)	0.006^*^
Relapses, *n* (%)	72 (36.7)	53 (40.0)	19 (47.5)	0.11
Interval time, month, median (IQR)	9.2 (6.3–15.2)	10.0 (6.3–15.0)	7.0 (6.3–12.0)	0.08
Follow-up time, month, median (IQR)	20.5 (13.4–37.1)	21.2 (15.0–40.4)	20.3 (11.6–36.4)	0.54

*Continuous variables were presented as mean ± SD or median (IQR = 25th−75th percentile), and categorical variables were described as percentages*.

*Normal-TG Group, patients whose triglyceride levels were below or equal to 1.7 mmol/L; High-TG Group, patients whose triglyceride levels were higher than1.7 mmol/L*.

*BMI, body mass index; TC, total cholesterol; TG, triglycerides; HDL, high density lipoprotein cholesterol; LDL, low density lipoprotein cholesterol; AQP4, aquaporin-4; EDSS, Expanded Disability Status Scale*.

* *p < 0.05*.

### Effects of Clinical Parameters on the Prognosis of NMOSD Patients

To explore potential factors that may affect the prognosis of patients, univariate logistic analysis was performed. As shown in Table 2, TG level was positively associated with an increased risk of poor recovery (OR 1.75, 95% CI 1.18–2.60, *P* = 0.005). Age of onset (OR 1.03, 95% CI 1.00–1.12, *P* = 0.024), AQP4 status (OR 1.44, 95% CI 1.02–2.04, *P* = 0.037), diabetes (OR 3.97, 95% CI 1.20–13.09, *P* = 0.049), statin treatment (OR 2.64, 95% CI 1.05–6.06, *P* = 0.04) initial EDSS (OR 1.59, 95% CI 1.29–1.90, *P* = 0.001), and relapses (OR 2.21, 95% CI, 1.17–4.18, *P* = 0.023) were significantly associated with outcomes in patients. Sex (OR 1.52, 95% CI 0.84–2.74, *P* = 0.17) and glucose level (OR 1.13, 95% CI 0.96–1.33, *P* = 0.15) had a moderate impact, but TC, HDL, LDL, BMI, hypertension, autoimmune diseases, and treatment methods were not associated with the outcomes. In addition, we observed a statistically significant correlation between TG level and relapse (OR 1.57,95% CI 1.07–2.31, *P* = 0.02).

**Table 2:** Univariate logistic regression analysis of factors associated with the outcomes of NMOSD patients.

**Variable**	**Outcomes**	
	**OR (95% CI)**	***P*-value**
Age of onset	1.03 (1.00–1.12)	0.024^*^
Sex	1.52 (0.84–2.74)	0.17
BMI	1.01 (0.92–1.12)	0.66
TC	1.09 (0.82–1.45)	0.56
TG	1.75 (1.18–2.60)	0.005^*^
HDL	0.63 (0.26–1.53)	0.31
LDL	1.20 (0.85–1.70)	0.38
Glucose level	1.13 (0.96–1.33)	0.15
^a^AQP4 status	1.44 (1.02–2.04)	0.037^*^
Hypertension	1.60 (0.55–4.65)	0.38
Diabetes	3.97 (1.20–13.09)	0.049^*^
Autoimmune disease	0.63 (0.13–3.05)	0.56
Corticosteroid treatment	0.68 (0.36–2.67)	0.43
Immunosuppressant treatment	0.91 (0.45–1.82)	0.7
Rehabilitation training	1.44 (0.67–3.06)	0.84
Statin treatment	2.64 (1.05–6.06)	0.04^*^
Initial EDSS	1.59 (1.29–1.90)	0.001^*^
Relapse	2.21 (1.17–4.18)	0.023^*^
^b^TG	1.57 (1.07–2.31)	0.02^*^

*BMI, body mass index; TC, total cholesterol TG, triglycerides; HDL, high density lipoprotein cholesterol; LDL, low density lipoprotein cholesterol; AQP4, aquaporin-4; EDSS, Expanded Disability Status Scale; OR, Odds ratio; CI, confidence interval*.

a Not-tested patients (n = 93) were excluded.

b The associations between TG level and relapses were analyzed.

* *p < 0.05*.

### Associations Between TG Level and Prognosis of NMOSD Patients According to Clinical Parameters

In order to further analyze the impact of various parameters on the association between TG level and prognosis, we subsequently divided the variables into different subgroups and conducted subgroup and interaction analysis. The results in Table 3 indicated that TG level had a positive association with poor outcomes according to all variables. Notably, we found significant heterogeneity between the subgroups analyzed according to BMI and corticosteroid treatment. As TG level increased, the risk of disability was obviously higher in patients with normal BMI (OR 4.90, 95% CI 2.10–11.44; *P* = 0.001) than those with high BMI (OR 0.93, 95% CI 0.20–3.40, *P* = 0.79). Compared to subjects without receiving corticosteroid treatment (OR 19.26, 95% CI 0.75–493.74, *P* = 0.07), those who treated with corticosteroid (OR 1.59, 95% CI 1.06–2.37, *P* = 0.024) had significantly lower chances of poor recovery because of elevated TG level.

**Table 3:** Association between TG and the outcomes according to baseline characteristics.

**Variables**	**Subgroup**	***N* (%)**	**Outcome**		
			**OR (95% CI)**	***P-*value**	**P for interaction**
Age (year)	≤60	167 (85.2%)	1.78 (1.17–2.70)	0.007^*^	0.93
	>60	29 (14.8%)	1.89 (0.53–6.72)	0.33	
Sex	Female	124 (63.3%)	1.41 (0.86–2.30)	0.17	0.11
	Male	72 (36.7%)	3.02 (1.27–7.17)	0.012^*^	
BMI (Kg/m^2^)	≤25	158 (80.6%)	4.90 (2.10–11.44)	0.001^*^	0.016^*^
	>25	38 (19.4%)	0.93 (0.20–3.4)	0.79	
TC (mmol/L)	≤5.0	162 (82.6%)	1.51 (0.97–2.33)	0.06	0.24
	>5.0	34 (17.3%)	2.77 (1.06–7.24)	0.04^*^	
HDL (mmol/L)	≤1.0	43 (21.9%)	1.45 (0.78–2.69)	0.24	0.49
	>1.0	153 (78.1%)	1.93 (1.14–3.28)	0.015^*^	
LDL (mmol/L)	≤3.0	134 (68.4%)	0.50 (0.23–1.06)	0.07	0.19
	>3.0	62 (31.6%)	2.58 (1.11–5.99)	0.028^*^	
Glucose (mmol/L)	≤6.1	147 (75%)	1.92 (1.19–3.11)	0.008^*^	0.69
	>6.1	49 (25%)	1.60 (0.75–3.39)	0.22	
Initial EDSS	≤3	60 (30.6%)	2.51 (1.20–5.65)	0.026^*^	0.32
	>3	136 (69.4%)	1.58 (1.00–2.50)	0.049^*^	
AQP4 status	Positive	64 (68.8%)	2.55 (1.63–3.83)	0.04^*^	0.14
	Negative	29 (31.2%)	1.43 (0.71–2.90)	0.32	
Hypertension	Yes	14 (7.1%)	2.76 (0.51–14.89)	0.24	0.54
	No	182 (92.9%)	1.67 (1.11–2.52)	0.014^*^	
Diabetes	Yes	9 (4.6%)	2.73 (0.21–35.56)	0.44	0.72
	No	187 (95.4%)	1.73 (1.16–2.57)	0.007^*^	
Autoimmune disease	Yes	10 (5.1%)	2.22 (0.68–7.26)	0.19	0.7
	No	186 (94.9%)	1.74 (1.14–2.66)	0.1	
Corticosteroid	Yes	180 (91.8%)	1.59 (1.06–2.37)	0.024^*^	0.042^*^
	No	16 (8.2%)	19.26 (0.75–493.74)	0.07	
Immunosuppressant treatment	Yes	56 (28.6%)	2.06 (1.04–4.06)	0.037^*^	0.58
	No	140 (71.4%)	1.63 (0.99–2.68)	0.06	
Rehabilitation training	Yes	38 (19.4%)	1.99 (0.87–4.54)	0.1	0.86
	No	158 (80.6%)	1.62 (1.02–2.55)	0.04^*^	
Statin treatment	Yes	21 (13.5%)	1.06 (0.4–2.85)	0.9	0.34
	No	175 (86.5%)	1.84 (1.20–2.83)	0.005^*^	
Relapse	Yes	72 (36.7%)	1.85 (1.06–3.21)	0.03^*^	0.6
	No	124 (63.3%)	1.41 (0.75–2.64)	0.28	
^a^TG (mmol/L)	≤3.1	156 (79.6%)	1.17 (0.46–2.99)	0.75	0.35
	>3.1	40 (20.4%)	2.03 (0.85–4.84)	0.11	

*BMI, body mass index; TC, total cholesterol; TG, triglycerides; HDL, high density lipoprotein cholesterol; LDL, low density lipoprotein cholesterol; CRP, C-reactive protein; AQP4, aquaporin-4; EDSS, Expanded Disability Status Scale; OR, Odds ratio; CI, confidence interval*.

a *The associations between TG level and relapses according to TG categories*.

* *p < 0.05*.

### TG Level Is a Predictor of the Outcomes of the First-Attacked NMOSD Patients

In the basic multivariate logistic regression modeling (Table 4), a higher TG level at study entry demonstrated a lower probability to regain good outcomes (OR 6.7, 2.12–21.18; *P* = 0.001). This association remained robust after adjusting for age of onset, sex, BMI, statin treatment initial EDSS, and relapses (OR 3.33;1.99–11.15; *P* = 0.02) in Adjusted I model. Subsequently, we further adjusted for BMI, glucose levels, complications and treatment methods in Adjusted-II model, but there still existed a statistically significant association between TG level and outcomes (OR 3.44; 1.02–11.64; *P* = 0.04).

**Table 4:** Adjusted logistic regression models of lipid parameters associated with the outcomes of NMOSD patients.

**Variable**	**Basic model^a^**		**Adjust-I model^b^**		**Adjust-II model^c^**	
	**OR (95% CI)**	***P*-value**	**OR (95% CI)**	***P*-value**	**OR (95% CI)**	***P*-value**
TG	6.70 (2.12–21.18)	0.001*	3.33 (1.99–11.15)	0.02*	3.44 (1.02–11.64)	0.04*
TC	1.98 (0.30–13.08)	0.48	7.38 (0.72–75.34)	0.09	7.57 (0.70–81.24)	0.1
HDL	0.75 (0.10–5.74)	0.78	0.29 (0.03–2.90)	0.29	0.39 (0.04–4.26)	0.44
LDL	0.24 (0.04–1.65)	0.24	0.06 (0.05–1.52)	0.21	0.07 (0.05–1.21)	0.14
Statin treatment			3.34 (0.58–19.10)	0.18	2.81 (0.47–16.89)	0.26
Age of onset			1.02 (0.07–1.06)	0.53	1.01 (0.96–1.05)	0.82
Sex			1.52 (0.51–4.55)	0.95	1.80 (0.54–6.05)	0.34
BMI			0.95 (0.80–1.13)	0.57	0.96 (0.79–1.15)	0.63
Initial EDSS			1.74 (1.26–2.42)	0.001*	1.90 (1.31–2.77)	0.001*
Relapse			3.81 (1.15–12.60)	0.03*	4.51 (1.24–16.43)	0.02*
Glucose level					1.05 (0.76–1.45)	0.77
Hypertension					0.47 (0.07–3.22)	0.47
Diabetes					5.81 (0.68–49.88)	0.11
Autoimmune disease					2.62 (0.20–34.82)	0.47
Corticosteroid treatment					1.24 (0.11–13.82)	0.86
Immunosuppressant treatment					1.36 (0.37–4.97)	0.64
Rehabilitation training					1.83 (0.45–7.38)	0.39

a *Basic model: Adjusted for TC, LDL, HDL*.

b Adjust-I model: Further adjusted for statin treatment, BMI, relapse, age, sex, and Initial EDSS.

c *Adjust-II model: Further adjusted for glucose level, hypertension, diabetes, autoimmune disease, corticosteroid, Rehabilitation, and immunosuppressant*.

*BMI, body mass index; TC, total cholesterol; TG, triglycerides; HDL, high density lipoprotein cholesterol; LDL, low density lipoprotein cholesterol; CRP, C-reactive protein; EDSS, Expanded Disability Status Scale; OR, Odds ratio; CI, confidence interval*.

## Discussion

NMOSD is a chronic autoimmune demyelinating disorder that generally leads to severe and irreversible disabilities including blindness and paralysis (22), but reliable prognostic indicators for this deleterious disease are still lacking. In this cohort study, we examined the possible associations of lipid parameters with disease progression through a large NMOSD dataset. The results indicated that TG level was positively correlated with clinical outcomes, and therefore, we speculated that TG level might have a prognosticative value for patients newly diagnosed with NMOSD. But levels of HDL, LDL, and TC did not relate to outcomes. To our best knowledge, this is the first report to demonstrate that high TG level may worsen disability in NMOSD.

As a key storage molecule of metabolic energy and fatty acids, TG is involved in many biological processes through mediation of lipogenesis or lipolysis, which are essential for organs to maintain normal function (23, 24). Emerging evidence indicate that TG metabolism pathways closely interacted with activated immune system (25–27). One possible mechanism was that excessive accumulation of TG caused metabolic disturbance and lipid deposition in lymphoid tissues, and those pathological conditions triggered immune activation (25). Additionally, TG was a leukocyte activator that stimulated inflammation by increasing the expression of antigen markers on surface of leukocytes (28). A recent clinical trial based on general US population suggested that elevated TG implied increased leukocyte profiles and lipid-lowering intervention might exert beneficial anti-inflammatory and immunomodulatory effects (29). In turn, inflammatory cytokines could also alter serum TG level (30). Previous studies showed that both tumor necrosis factor and interleukin-l could induce a remarkable increase in serum TG level during infection and inflammation process (31).

Among autoimmune demyelination diseases of the CNS, patients with MS and NMO had higher serum TG levels than healthy controls (32). Elevated TG levels was able to increase IL-6 concentrations in cerebrospinal fluid (16) and augment the risk of subsequent relapse in participants after a first demyelinating event (33), while lower serum TG level and BMI could ameliorate central inflammation and reduce the accumulation of disability in relapsing-remitting MS patients (21). During the pathological process of MS, higher lipoproteins can aggravate inflammation at vascular endothelium, leading to immune cells across the activated endothelium of blood brain barrier (14). There is no data on how TG plays a role in the pathogenesis and development of NMOSD. NMOSD was considered as a severe variant of MS, and various types of immune cells [including regulatory T cells (34), neutrophils (35), and B lymphocytes (36)] and cytokines [including IL-6 (37), IFN (38), and IL-10 (34)] are involved in disease onset and progression. It has been proposed that CNS inflammation mediated by cytokines may exacerbate the clinical status of NMO (39). Therefore, we speculated that TG might indirectly reflect the inflammatory status by interacting with immune responses in NMOSD patients, but this assumption needs further research.

In our study, patients who were newly diagnosed with NMOSD were eligible, allowing us to eliminate the effects of previous treatment on outcome events. All subjects were divided into two groups according to TG level at admission. We found that patients with high TG level (≥1.7 mmol/L) were more likely to have increased TC and LDL values, while the HDL levels significantly decreased (Table 1), which indicated that dyslipidemia also exists in patients with NMOSD. This finding was in line with a case-control study regarding lipoproteins in NMO showing that NMO patients have elevated TC, TG, and LDL levels compared with those in healthy controls (32).

The average age of onset was 43.4 years in our study, which is comparable another previous study regarding epidemiology of NMOSD in Sweden (19). A recent research suggested that age of onset may be a prognostic factor for NMOSD, in which lower aged patients with NMOSD had a higher chance of good recovery after receiving immunoadsorption treatment (40). In line with this research, our univariate analysis also found that age of onset was positively associated with outcomes in first-attacked NMOSD patients. AQP4-IgG was a specific biomarker for the diagnosis of NMO, which can cause demyelination and neurologic deficit by binding to astrocytes and leading to astrocyte injury (41). Positive AQP4 status was closely related to rapid progress, severe tissue damage, and abundant infiltration of leukocyte in some NMO patients (42). Consistent with results of previous studies, our results suggested that AQP4 status had positive correlations with outcomes of NMOSD cases. No significant association between BMI and outcomes was observed in our study, while a cohort study showed that BMI was a predictive factor of EDSS worsening for patients with refractory NMO who were treated with rituximab, but only 21 subjects were included in this research (20). Statin has been reported to have anti-inflammatory and immunomodulatory effects in an experimental mouse model of NMO by increasing CD55 expression (43), and our data also support that statin treatment on the disease courses has a significant correlation to outcomes. Since long-term disability depends on initial symptoms and the accumulation of disability after several attacks (21, 44, 45), we also examined the correlations among initial EDSS, relapse, and outcomes. Accordingly, our findings proved that the initial EDSS and relapses can affect the prognosis of patients, suggesting the importance of early prevention and effective treatment. Previous evidence also suggested that TG level showed significant correlation with relapses in MS (14); therefore, we examined whether this association existed in patients with NMOSD. Consistent with results of the previous research, our results indicated that high TG level increased the risk of relapse. In our cohort, the risk for first relapse was highest in the first year after disease onset (median 9.2 months) (19), while this figure was slightly larger with a median 1.42 year in a 26-year nationwide population-based study, which we attributed to the relatively short follow-up time in our study.

A matched case–control study of NMO suggested that a lower BMI was associated with a higher mortality in women (46), and one of the potential mechanisms hypothesized by this research was that a lower BMI could argument the susceptibility to autoimmunity because of the deregulated level of adipokines or sex hormones (47, 48). Interestingly, our subgroup and interaction analysis showed that patients with normal BMI displayed a dramatically higher risk of adverse outcomes than those with higher BMI as TG level elevated, but the mechanisms about how BMI interacts with the TG biological pathway and worsen disease progression in NMOSD still unclear. Since only 38 patients were included in the higher BMI group, the results may be biased and need to be further verified in a larger sample size study.

Some limitations of our study need to be addressed further. Because the total number of subjects in our cohort study was small and patients were from a single center with a relatively short follow-up duration, the results need to be further validated in larger, multicenter, and long follow-up duration studies. Previous studies suggested that low dairy consumption and lack of physical activity were potential risk factors for NMOSD (9, 49), but we failed to include these two factors in our retrospective study due to the majority of patients providing inaccurate data on their daily diet and exercise habits, which may bias our results. In addition, it is essential to explore the role of TG pathway in the progression of NMOSD. In conclusion, our study suggests that a significant association exists between TG level and outcomes in NMOSD, and early monitoring and treatment of adverse lipid profiles in NMOSD may be necessary.

## Data Availability Statement

The datasets generated for this study are available on request to the corresponding author.

## Ethics Statement

The studies involving human participants were reviewed and approved by the Ethics Committee of Zhengzhou University. The patients/participants provided their written informed consent to participate in this study.

## Author Contributions

KW and QH were responsible for conceiving and designing the study and drafted the manuscript. LW, RD, and YL conducted the regular follow-up of all cases. YJ and JT undertook the task of assessing the initial EDSS and residual EDSS scores of all patients. YY, QH, KW, and LJ conducted the data analysis. All authors were involved in the process of data collection, reviewed, and approved the final submitted manuscript.

### Conflict of Interest

The authors declare that the research was conducted in the absence of any commercial or financial relationships that could be construed as a potential conflict of interest.
